# Accuracy of Magnetic Resonance Imaging in Diagnosis of Anterior Cruciate Ligament and Meniscus Tear: An Observational Study

**DOI:** 10.31729/jnma.8982

**Published:** 2025-05-31

**Authors:** Sushil Thapa, Sunil Panta, Aarati Adhikari, Hari Prasad Upadhyay, Sarik Kumar Shrestha

**Affiliations:** 1 Consultant Orthopaedic Surgeon, Department of Orthopedics and Trauma Surgery, Bharatpur Hospital, Bharatpur, Chitwan, Nepal; 2 Consultant Radiologist, Department of Radiology, College of Medical Sciences, Bharatpur, Chitwan, Nepal; 3 Assistant Professor of Statistics, Birendra Multiple Campus, Chitwan, Nepal

**Keywords:** *accuracy*, *anterior cruciate ligament*, *magnetic resonance imaging*, *meniscus*, *tear*

## Abstract

**Introduction:**

Magnetic resonance imaging is used for the diagnosis of anterior cruciate ligament and meniscus tears but its value in the diagnosis of these pathologies is limited. This study aims to evaluate the diagnostic value of magnetic resonance imaging in diagnosing anterior cruciate ligament and meniscus tears.

**Methods:**

This was an observational cross-section study conducted at a tertiary-level hospital in Nepal. The patients operated on for anterior cruciate ligament and/or meniscus tear were considered for the study. The accuracy of magnetic resonance imaging was calculated by comparing its findings with those in arthroscopy.

**Results:**

The total number of cases were 134. The sensitivity and specificity were as follows: medial meniscus 88.81%, 95.10%; lateral meniscus 57.12%, 95.14%; anterior cruciate ligament 96.12%, 75%. The sensitivity and specificity of the injuries for 3 Tesla vs 1.5 Tesla Magnetic Resonance Imaging were as follows: medial meniscus 89.20%, 97.84% vs 87.52%, 87.52%; lateral meniscus 65.25%, 100% vs 30%, 90.91%; anterior cruciate ligament 96.12%, 75% vs 96.12, 75%.

**Conclusions:**

The sensitivity of magnetic resonance imaging in diagnosing anterior cruciate ligament injury was higher while specificity was higher for lateral meniscus injury. The study also showed that 3 Tesla was better than 1.5 Tesla magnetic resonance imaging in diagnosing meniscus injury.

## INTRODUCTION

Anterior cruciate ligament (ACL) and meniscus injuries are common injuries and ACL tears are associated with meniscus tears in 50% of the cases.^1^ ACL and meniscus serve important functions in the knee joint^2^. Magnetic Resonance Imaging (MRI) is often considered the gold standard for diagnosis of ACL and meniscus tears but its value in diagnosis of these pathologies is limited.^3-5^

The sensitivity of MRI is lower for meniscus tears in ACL-deficient knees and varies with different parts of the meniscus.^6,7^ MRI has lower sensitivity for lateral meniscus (LM) tears due to magic angle phenomenon, popliteus tendon, meniscofemoral ligament and pulsation artefact.^8-10^ There are only a few studies discussing the accuracy of MRI based on its strength.

The primary objective of this study was to evaluate the diagnostic value of MRI for ACL and meniscus tear. The secondary objective was subgroup analysis of accuracy of MRI of different strengths.

## METHODS

This observational cross-section study was conducted at Bharatpur Hospital, Chitwan, Nepal, a tertiary-level hospital. The study duration was from January 13, 2021, to January 12, 2024. Ethical approval was obtained from the Institutional Review Committee (IRC) of Bharatpur Hospital (Reference number: 077/78006) before commencing the study.

Ihe sample size was calculated using the prevalence, sensitivity, and specificity of MRI in diagnosing ACL tears based on previous studies.^3,7^ The sample size obtained was 134 based on sensitivity and 18 based on specificity. A similar process was followed for MRI in diagnosing medial meniscus tears. Considering values from previous studies,^3,7^ the sample size obtained was 43 based on sensitivity and 58 based on specificity. Finally, the prevalence, sensitivity, and specificity of MRI in diagnosing lateral meniscus tears.^3,7^ were used to determine the sample size. This resulted in 89 samples based on specificity and zero samples based on sensitivity, as the sensitivity of MRI for diagnosing lateral meniscus tears was 100%. The highest calculated sample size, which was 134, was considered the final sample size.

The clinical diagnosis of ACL tear was made from positive results of Lachmann test. The clinical diagnosis of meniscus tear was made from medial/lateral joint line tenderness with positive results of McMurray's test. Radiological diagnosis of ACL and meniscus tear was made from the MRI findings. We included patients with clinical and radiological evidence of ACL tear and/ or meniscus tear scheduled for ACL reconstruction and/or meniscus surgery. The exclusion criteria were associated fractures, ramp tears, root tears, grade II meniscus tears and history of previous knee surgery. Before enrolling the patient into study, a written consent was taken from the patients. All the patients' particulars, laterality, type of tear, magnetic strength of MRI and arthroscopy picture were recorded in data collection tool. This data was then copied to an Excel spreadsheet.

The MRI imaging was performed using either of the following two systems: 3T (3 Tesla) system (Siemens) or 1.5T (1.5 Tesla) system (Siemens), (Table 1). A 15-channel phased-array knee coil was used. Radiologists and fellowship-trained orthopaedic surgeons assessed the MRI films. In certain instances, when there was disagreement on the evaluation made by radiologists, a group consensus between them and surgeons was made. When an ACL was not visualized, it was considered torn. When some remnants of the ligament were preserved, two factors were considered: firstly, a signal change in the fibers and secondly an angle between ACL-Blumensaat line.^11^ When an ACL had a hyperintense signal change and apex of ACL-Blumensaat line angle directed towards the femur, the ACL was considered normal (Table 1). If this angle directed towards tibia, it was considered torn. In the MRI, the meniscus was considered torn if a hyperintense signal change extended up to the articular surface. While intra-substance signal change confined within a substance was considered degeneration.

These findings in MRI about ACL and meniscus tears were compared with the findings in arthroscopy.

**Table 1:** MRI protocols for Anterior Cruciate Ligament and Meniscus Tear.

3T MRI			
Parameters	T1	Intermediate	T2
TR/TE	620/12 ms	3560-3670/21 ms	3000/17-86 ms
Slice thickness	3mm	3mm	3mm
Turbo factor	7	5	5
Field of view	160 mm	160 mm	160 mm
Matrix	307 X 384	307 X 384	307 X 384
Distance factor	10%	10%	10%
1.5T MRI			
Parameters	T1	Intermediate	T2
TR/TE	555/15 ms	3470-3590/28 ms	3340/21-85 ms
Slice thickness	4mm	4mm	4mm
Turbo factor	5	5	5
Field of view	160 mm	160 mm	160 mm
Matrix	307 X 384	307 X 384	307 X 384
Distance factor	10%	10%	10%

MRI=Magnetic Resonance Imaging; ST=Slice thickness; TF = Turbo Factor; FOV=Field of View; DF=Distance Factor;3T= 3 Tesla 1.5 T=1.5 Tesla

After administering spinal anaesthesia, the patient was kept in supine position. A 4mm, 30-degree scope was used. A fellowship-trained orthopaedic surgeon with specialization in sports medicine did the exploration of the ACL and meniscus. An anterolateral portal was made and systematic diagnostic arthroscopy done. The pathology of ACL and meniscus noted. If ACL tear was detected, the hamstring graft was harvested and prepared. An anteromedial portal was made and lateral and medial menisci were explored. If a lateral meniscus tear was not clearly visualized, the arthroscope was introduced through the posterolateral portal and further assessment done. The meniscus procedure (repair or excision) was completed if required. The ACL reconstruction was done if required using either a hamstring autograft or peroneus longus autograft.

The findings in MRI and those in arthroscopy were recorded in the data collection tool. A single tool contained an entire information about a patient. The accuracy and completeness of data were verified. Collected data was entered into MS Excel spreadsheet and then imported into IBM SPSS Statistics for Windows, version 244 (IBM Corp., Armonk, N.Y., USA) for subsequent analysis. Continuous variables were presented as means and categorical variables were presented as percentages and counts. The sensitivity, specificity, positive predictive value (PPV) and negative predictive value (NPV) of the MRI for ACL tear, medial meniscus tear and lateral meniscus tear were calculated with their respective 95% CI. To calculate the sensitivity, specificity, PPV and NPV of MRI for ACL and meniscus tears, findings on arthroscopy was considered as the gold standard for diagnosis.^3,5^ True positive was defined as presence of tear on both arthroscopy and MRI and true negative was defined as absence of tear on both arthroscopy and MRI. False positive was defined as presence of tear on MRI but no tear found during arthroscopy and false negative was defined as absence of tear on MRI but tear visualized during arthroscopy.

These parameters were calculated for 3T and 1.5T MRI separately as well. The sensitivity, specificity, PPV and NPV of 3T MRI and 1.5T MRI for diagnosis of ACL tear and meniscus tear were compared. Additionally, the sensitivity, specificity, PPV and NPV of MRI for diagnosis of medial meniscus tear and lateral meniscus tear was also compared using the McNemar test. Significance was defined as p-value of less than 0.05.

## RESULTS

The study group consisted of 134 patients, where 91 (67.91%) were male, and 88 (65.55%) were in the age group of 20-40 years. There were 103 (76.86%) imaging done with 3 Tesla MRI. Of all the patients, 81 (60.45%) had meniscal tear of which 24 (29.63%) had longitudinal and bucket handle tears and there were 125 (93.28%) ACL tears. Of all the patients, 90 (67.16%) were operated within 30 days of the imaging. (Table 2).

**Table 2:** Demography of patients undergoing MRI for Anterior Cruciate Ligament and Meniscus Tear (n=134).

Particulars		Frequency	Percentage (%)
Age	<20 years	14	10.45
	20-40 years	88	65.67
	>40 years	32	23.88
Sex	Male	91	67.91
	Female	43	32.09
MRI	3 T	103	76.87
	1.5 T	31	23.13
Meniscus			
Meniscus tear		81	60.45
Longitudinal Tear		24	29.63
Bucket Handle Tear		24	29.63
Radial Tear		10	12.34
Complex Tear		23	28.39
Normal		53	39.55
ACL			
ACL tear		125	93.28
Normal		9	6.72
Average Injury to Imaging duration (days)		427.30 days		
Imaging to Surgery duration (days)			
1-30		90	67.16
31-60		25	18.66
>60		19	14.18

MRI = Magnetic Resonance Imaging; ACL = Anterior Cruciate Ligament

**Table 3:** Comparison of diagnostic accuracy with 3T and 1.5T MRI in patient with Anterior Cruciate Ligament and Meniscus Tear (n=134).

Category	Parameters	All Patients	3 T	1.5 T	P Value
	Sensitivity	88.81%	89.20%	87.52%	0.80
Medial meniscus	Specificity	95.10%	97.84%	87.52%	0.09
	PPV	92.33%	98.03%	87.52%	0.08
	NPV	83.33%	88.24%	87.52%	0.91
	Sensitivity	57.12%	65.25%	30%	<0.01
Lateral meniscus	Specificity	95.14%	100%	90.91%	0.04
	PPV	95.52%	100%	60%	<0.001
	NPV	83.33%	86.40%	74.07%	0.14
	Sensitivity	76.99%	80.66%	65.30%	<0.001
Both menisci	Specificity	96.81%	99.12%	89.44%	0.04
	PPV	94.42%	98.66%	80.94%	0.012
	NPV	85.21%	87.14%	79.06%	0.31
	Sensitivity	96.12%	96.12%	96.12%	1
	Specificity	75%	75%	75%	1
ACL	PPV	98.40%	98.40%	98.40%	1
	NPV	45.44%	45.44%	45.44%	1

T=Tesla; PPV = Positive predictive value; NPV = Negative predictive value; MRI=Magnetic Resonance Imaging;

The sensitivity and specificity of MRI to detect medial meniscus tear was 88.81% and 96.10% and that for lateral meniscus tear was 57.12% and 95.14%. The sensitivity for diagnosing medial meniscus tear with 3T MRI and 1.5T MRI was 89.20% and 87.52% (p=<0.01) and the specificity was 97.84% and 87.52% (p=0.09). Similarly, sensitivity for diagnosing lateral meniscus tear with 3T MRI and 1.5T MRI was 65.25% and 30% (p=0.80) and the specificity was 100% and 90.01%. (p=0.04). The sensitivity and specificity MRI to detect ACL injury it was 96.12% and 75%. The sensitivity for diagnosing ACL tear with 3T MRI and 1.5T MRI was 96.12% and 96.12% (p=1) and the specificity was 75% and 75% (p=1). (Table 3).

## DISCUSSION

This study demonstrated that the sensitivity of MRI was good for detecting anterior cruciate ligament injuries, moderate for medial meniscus tear and low for lateral meniscus tear. The sensitivity and specificity of MRI also varied with strength of MRI. The present study concluded that MRI had highest sensitivity for ACL (96.12%) and highest specificity for medial meniscus tear (96.10%). This is in contrast to a meta-analysis by Phelan et al. which estimated 87% sensitivity for ACL tears.^12^ In another study by Zhao et al., the sensitivity and specificity of MRI in the diagnosis of ACL injury were 95.45% and 91.67% respectively.^13^ They considered an interruption of ACL continuity and thickening with edema as the direct signs of ACL tear. In present study, besides these two direct signs, another parameter; ACL-Blumensaat line angle was used. This line is particularly helpful when ACL fibers are apparent, but it is not conclusive about tear. The use of this additional parameter probably increased the sensitivity for diagnosing ACL tear in the present study.

In the present study, the sensitivity and specificity of MRI for diagnosing medial meniscus tears were 88.81% and 95.10% respectively for medial meniscus tears; 57.12% and 95.14% respectively for lateral meniscus tears. This finding is similar to a study by Joshi et al. where the sensitivity of MRI for lateral meniscus tear (68.2%) is significantly lower than that for medial meniscus tear (92.9%).^6^ In a systematic review by Crawford et al., the sensitivity and specificity for medial and lateral menisci were 91.4% and 76 % respectively.^5^ In a study by Phelan et al., the summary estimates of sensitivity and specificity of MRI were 89 % and 88% respectively for medial meniscus tears; and 78% and 95% respectively for lateral meniscus tears.^12^ In another retrospective study by Dawkins et al., the sensitivity and specificity of MRI for lateral meniscus tears were 51% and 86.5% respectively while that for medial meniscus tears were 83.2% and 80.6% respectively.^14^ All the above-mentioned studies have concluded that the diagnostic accuracy of MRI is better for medial meniscus as compared to that for lateral meniscus which is similar to the finding of the present study. These investigations have not assessed how an MRI's magnetic field strength affects precision.

In a stratified analysis, 3T MRI was more sensitive (80.66%) (p<0.001) and specific (99.12%) (p=0.04) in detecting meniscus tear. This finding is different from a study by Phelan et al.^12^ who concluded that magnetic field strength had no significant effect on sensitivity and specificity in diagnosing meniscus tear. The results in a meta-analysis by Qi et al. indicated that both 3T and 1.5 T MRI offer high sensitivity for meniscus tears.^15^ The present study has demonstrated higher sensitivity (65.25%, p<0.001) and specificity (100%, p=0.04) to diagnose lateral meniscus tear with 3T MRI. A study by Hancock et al. concluded that there is no clear disadvantage of 1.5 T imaging compared to 3T in paediatric knees.^16^ In these studies, the MRI from several centres were considered. While in the present study, 3T and 1.5T MRI from two respective centres only, were considered. The different scanners with similar strength MRI may show variable accuracy because of several other confounding elements. This could be a part of the reason why the present study does not corroborate with the above-mentioned studies.

A study by Let al. evaluated 97 patients and concluded that the sensitivity of MRI was good for ACL injuries (87.6%).^17^ This finding is similar to that of the current study. Kim et al. studied 544 patients and compared the 3T MRI findings with those in arthroscopy. They found that the sensitivity of MRI in detecting the medial meniscus tear and lateral meniscus tear was 91.8% and 80.8% respectively.^18^ The conclusion of the present study is similar to their findings. Another study by Zhao et al. among 77 participants showed that the sensitivity and specificity of MRI in the diagnosis of ACL tear was 95.45% and 91.67% respectively.^19^ This study has similar sensitivity but better specificity of MRI for ACL tear as compared to the current study. They have studied only 1.5 T MRI and have not mentioned the diagnostic criteria for ACL tear.

In an evaluation of 320 patients by Razak et al., MRI was 100% sensitive in diagnosing ACL injuries while it was 77% and 57% sensitive in the diagnosis of MM and LM injuries respectively.^20^ The present study is not consistent with these findings. They have not set the diagnostic criteria for diagnosis of ACL tear and meniscus tear. In most of the above studies, variable strength MRIs were not included and the diagnostic criteria were not been defined in contrast to the present study.

There are a few limitations of this study. The surgeon who also evaluated the patient interpreted some MRIs. This is a potential source of bias. Some patients, who are physically more active or who encounter additional trauma may sustain further tears or may aggravate the existing ones after the last MRI. Improvement of MRI sequence, use of specific knee coils and scanning techniques are confounding factors that affect the accuracy of MRI. The duration of imaging to surgery also affects the diagnostic value. We also recommend future studies on comparison between variable strengths MRI on a larger sample.

## CONCLUSION

The sensitivity of MRI in diagnosing ACL injury was higher while specificity was higher for medial and lateral meniscus injury. Between medial and lateral meniscus injury, the sensitivity was higher for medial meniscus while specificity was equal for both menisci. The study also showed that 3T MRI was better than 1.5 T MRI in diagnosing lateral meniscus injury however, there was no difference between those MRI for diagnosing ACL injury.

## References

[1] Feucht MJ, Bigdon S, Bode G, Salzmann GM, Dovi-Akue D, Sudkamp NP (2015). Associated Tears of the Lateral Meniscus in Anterior Cruciate Ligament Injuries: Risk Factors for Different Tear Patterns.. J Orthop Surg Res..

[2] Grassi A, Dal Fabbro G, Di Paolo S, Stefanelli F, Macchiarola L, Lucidi GA (2019). Medial and Lateral Meniscus Have a Different Role in Kinematics of the ACL-Deficient Knee: A Systematic Review.. J ISAKOS..

[3] Behairy NH, Dorgham MA, Khaled SA (2009). Accuracy of Routine Magnetic Resonance Imaging in Meniscal and Ligamentous Injuries of the Knee: Comparison With Arthroscopy.. Int Orthop..

[4] Fox MG (2007). MR Imaging of the Meniscus: Review, Current Trends, and Clinical Implications.. Radiol Clin North Am..

[5] Crawford R, Walley G, Bridgman S, Maffulli N (2007). Magnetic Resonance Imaging Versus Arthroscopy in the Diagnosis of Knee Pathology, Concentrating on Meniscal Lesions and ACL Tears: A Systematic Review.. Br Med Bull..

[6] Joshi A, Singh N, Basukala B, Bista R, Tripathi N, Pradhan I (2021). Accuracy of Magnetic Resonance Imaging for Meniscal Body Tear in Anterior Cruciate Ligament-Deficient Knees Compared to Anterior Cruciate Ligament-Intact Knee.. J Arthrosc Surg Sport Med..

[7] Gyawali B, Joshi A, Kayastha N (2020). Knee Injuries: Correlation of MRI With Arthroscopic Findings.. J Patan Acad Heal Sci..

[8] Sharifah MIA, Lee CL, Suraya A, Johan A, Syed A, Tan SP (2015). Accuracy of MRI in the Diagnosis of Meniscal Tears in Patients With Chronic ACL Tears.. Knee Surg Sports Traumatol Arthrosc..

[9] De Smet AA, Mukherjee R (2008). Clinical, MRI, and Arthroscopic Findings Associated With Failure to Diagnose a Lateral Meniscal Tear on Knee MRI. Am J Roentgenol..

[10] Peterfy CG, Janzen DL, Tirman PF, van Dijke CF, Pollack M, Genant HK (1994). "Magic-Angle" Phenomenon: A Cause of Increased Signal in the Normal Lateral Meniscus on Short-TE MR Images of the Knee.. AJR Am J Roentgenol..

[11] Adhikari V, Joshi A, Singh N, Pradhan I (2020). Predictive Accuracy of Blumensaat Line Angle and Its Apex Along With Anterior Cruciate Ligament Inclination Angle for Diagnosis of Anterior Cruciate Ligament Tear With Abundant Remnant..

[12] Phelan N, Rowland P, Galvin R, O'Byrne JM (2016). A Systematic Review and Meta-Analysis of the Diagnostic Accuracy of MRI for Suspected ACL and Meniscal Tears of the Knee.. Knee Surg Sports Traumatol Arthrosc..

[13] Zhao M, Zhou Y, Chang J, Hu J, Liu H, Wang S (2020). The Accuracy of MRI in the Diagnosis of Anterior Cruciate Ligament Injury.. Ann Transl Med..

[14] Dawkins BJ, Kolin DA, Park J, Fabricant PD, Gilmore A, Seeley M (2022). Sensitivity and Specificity of MRI in Diagnosing Concomitant Meniscal Injuries With Pediatric and Adolescent Acute ACL Tears.. Orthop J Sport Med..

[15] Cheng Q, Zhao F-C (2018). Comparison of 1.5- and 3.0-T Magnetic Resonance Imaging for Evaluating Lesions of the Knee: A Systematic Review and Meta-Analysis (PRISMA-Compliant Article).. Medicine (Baltimore)..

[16] Hancock GE, Hampton MJ, Broadley P, Ali FM, Nicolaou N (2021). Accuracy of Magnetic Resonance Imaging of the Knee for Intra-Articular Pathology in Children: A Comparison of 3T Versus 1.5 T Imaging.. J Arthrosc Jt Surg..

[17] Li X, Hou Q, Zhan X, Chang L, Ma X, Yuan H (2022). The Accuracy of MRI in Diagnosing and Classifying Acute Traumatic Multiple Ligament Knee Injuries.. BMC Musculoskelet Disord..

[18] Kim SH, Lee HJ, Jang YH, Chun KJ, Park YB (2021). Diagnostic Accuracy of Magnetic Resonance Imaging in the Detection of Type and Location of Meniscus Tears: Comparison With Arthroscopic Findings.. J Clin Med..

[19] Zhao M, Zhou Y, Chang J, Hu J, Liu H, Wang S, Si D, Yuan Y, Li H (2020). The Accuracy of MRI in the Diagnosis of Anterior Cruciate Ligament Injury.. Ann Transl Med..

[20] Abd Razak HRB, Sayampanathan AA, Koh TH, Tan HC (2015). Diagnosis of Ligamentous and Meniscal Pathologies in Patients With Anterior Cruciate Ligament Injury: Comparison of Magnetic Resonance Imaging and Arthroscopic Findings.. Ann Transl Med..

